# The usefulness of chief complaints to predict severity, ventilator dependence, treatment option, and short-term outcome of patients with Guillain-Barré syndrome: a retrospective study

**DOI:** 10.1186/s12883-017-0982-3

**Published:** 2017-11-21

**Authors:** Ying Wang, Pei Shang, Meiying Xin, Jing Bai, Chunkui Zhou, Hong-Liang Zhang

**Affiliations:** 1 Neuroscience Centre, Department of Neurology, the First Hospital of Jilin University, Xinmin Street 71#, Changchun, 130021 China; 2 Current address: Department of Life Sciences, the National Natural Science Foundation of China, Beijing, China

**Keywords:** Guillain-Barré syndrome, Chief complaint, Retrospective study, Clinical manifestations, Disease severity

## Abstract

**Background:**

It remains an urgent need for early recognition of disease severity, treatment option and outcome of Guillain-Barré syndrome (GBS). The chief complaint may be quickly obtained in clinic and is one of the candidates for early predictors. However, studies on the chief complaint are still lacking in GBS. The aim of the study is to describe the components of chief complaints of GBS patients, and to explore association between chief complaints and disease severity/treatment option/outcome of GBS, so as to aid the early prediction of the disease course and to assist the clinicians to prescribe an optimal early treatment.

**Methods:**

A total of 523 GBS patients admitted to the First Hospital of Jilin University from 2003 to 2013 were enrolled for retrospective analysis. The data of chief complaints, clinical manifestations, and treatment options, etc. were collected. The clinical severity was evaluated by the Medical Research Council sum score and the Hughes Functional Grading Scale. The prognosis at 6 month after discharge was described by modified Erasmus GBS outcome score. The clinic GBS severity evaluation scale (CGSES), a newly established model in our study, was used to explore the role of chief complaints to predict intravenous immunoglobulin (IVIg).

**Results:**

The major components of the chief complaints of GBS patients were weakness, numbness, pain, cranial nerve involvement, dyspnea, ataxia and autonomic dysfunction. Chief complaint of weakness was a predictor of severe disease course and poor short-term outcome, while chief complaint of numbness and cranial nerve involvement were promising predictors. Cranial nerve involvement was the predictor of ventilator dependence. The percentages of 366 GBS patients, who need IVIg treatment at nadir with CGSES ranging from 1 to 4, were 50.00, 67.34, 80.61, and 90.67%, respectively.

**Conclusions:**

Chief complaints are clinic predictors of disease severity, ventilator dependence and short-term outcome. IVIg treatment during hospitalisation could be predicted in clinic using CGSES score.

## Background

Most of the studies on the clinical features of Guillain-Barré syndrome (GBS) are based on objective parameters including medical history, physical examinations, laboratory examinations and electrophysiological examinations, etc. [1]. However, chief complaints, the most significantly subjective experience on the disease that drive patients to doctors have not received equal focus of attention [1, 2]. The importance of the chief complaint is emphasised in some of diseases [3]. For example, subjective cognitive impairment is the definition given to the subjects whose results on neuropsychological tests are normal but with complaints of memory impairment; a growing body of evidence indicates that subjective cognitive impairment could be a pre-clinical phase of Alzheimer’s disease [3]. The association between the chief complaint of patients with GBS and the severity/prognosis of disease remains unknown. A systematic study of the chief complaint of GBS is warranted for the following reasons. Firstly, chief complaints, the most significant symptoms reported by patients are not always in parallel with the results of physical examinations [4, 5]. Empirically, for most of the time chief complaints reflect the most significant part of the clinical manifestations, and for some time, chief complaints contain symptoms that could not be found by physical examinations. For example, an 82-year-old woman complained of back pain and glove-and-stocking paraesthesia for 5 days, but her neurological examination could be unremarkable [5]. Secondly, the chief complaint is potentially an early predictor of the severity, treatment option and prognosis of GBS. GBS is a devastating disease mainly characterised by progressively symmetrical flaccid paralysis [1, 2]. In severe cases, patients may develop respiratory muscle paralysis and need intensive care [1, 2]. Thus, early prediction of the disease course could assist clinicians to provide an optimal management to the patients at admission. For example, if a patient has a high risk to develop respiratory muscle paralysis at nadir, a respiratory monitor could be used at admission. Currently, the chief complaint is not on the list of predictors for GBS. The prognostic factors for death in GBS patients are age, the severity of disease and the speed of progression [6]. For the survivors, various predictors of prognosis have been addressed, such as an older age, severe disability at admission, cranial nerve involvement, ventilator dependence, autonomic dysfunction, neck flexor weakness and acute motor axonal neuropathy, and so forth [7–9]. Moreover, a clinical prognostic scoring model has been established to depict the outcome of GBS [10]. Age, antecedent diarrhea, and Hughes Functional Grading Scale (HFGS) score at 2 weeks after entry have been identified as predictors for the outcome of GBS 6 month after discharge [10].

The chief complaint is the first information that physicians obtain from patients, and it may assist clinicians to make an early recognition of the severity and prognosis of the disease. However, chief complaints are rarely taken into account when the predictors for severity/prognosis of GBS are explored [6–10]. Herein, we retrospectively investigate the components of chief complains so as to further explore their role in predicting disease severity, treatment option and prognosis.

## Methods

**Fig. 1:**
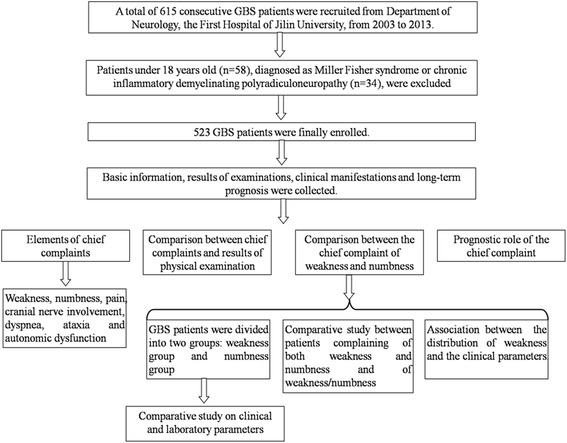
Flow chart of subject enrollment. This investigation was on the base of the clinical information of 615 consecutive Guillain-Barré syndrome (GBS) patients. Patients with younger age, Miller Fisher syndrome or chronic inflammatory demyelinating polyradiculoneuropathy were excluded. The association between the chief complaint and the severity as well as prognosis was explored. The elements of the chief complaint were depicted, and the differences between GBS patients with distinct chief complaints were studied

The aim of the study is to explore the important role of the chief complaint in GBS, and the study design is showed in Fig. 1.

### Study subjects

A total of 615 consecutive GBS patients were enrolled from Department of Neurology, the First Hospital of Jilin University, during the time interval between 2003 and 2013 (Fig. 1). Thirty-four patients, with the diagnosis of Miller Fisher syndrome or later identified chronic inflammatory demyelinating polyradiculoneuropathy, were excluded. Fifty-eight subjects with the age under 18 years old were also ruled out due to their different clinical characteristics from adult patients [11]. Finally, a cohort of 523 GBS patients fulfilling the inclusion criteria [12] and contradictive to the exclusion criteria were included for data analysis. For these patients, data of demographics, chief complaints, antecedent infection, history of past illness, clinical manifestations, results of blood and cerebrospinal fuild examinations, results of electrophysiological study and ventilation dependence were subsequently collected. The short-term prognosis was described by HFGS at discharge, and by the modified Erasmus GBS outcome score (mEGOS), ranging from 0 to 9, at 6 months after discharge [10].

### Measurement of disability

The clinical severity and functional impairment of GBS patients were assessed by two widely used scales, i.e. the Medical Research Council (MRC) sum score and HFGS score. The MRC sum score, ranging from 0 (tetraplegic) to 60 (normal strength), described the strength of six pairs of symmetrical muscles in both upper and lower extremities [13]. The definition of the HFGS score was that, 0: healthy state; 1: minor symptoms and capable of running; 2: able to walk 5 m or more without assistance but unable to run; 3: able to walk 5 m across an open space with help; 4: bedridden or chair-bound; 5: requiring assisted ventilation for at least part of the day; 6: dead [14]. The nadir of the disease course was identified as the day during hospitalisation when the lowest MRC sum score was observed.

### The clinic GBS severity evaluation scale (CGSES)

A model, namely CGSES, for predicting the disease severity and guiding the treatment option during hospitalisation was established based on the information that could be easily obtained in clinic after a quick inquiry. Finally, four of the clinical parameters, which could predict the progress of GBS during hospitalisation, were quantified to build CGSES, including duration between onset to admission, chief complaints of weakness, numbness and cranial nerve involvement. The data of 373 GBS patients recruited from 2003 to 2011 were employed to establish the CGSES, and the data of the other 150 patients admitted from 2012 to 2013 were utilised to validate the model. The severe GBS patients, among whom the immunotherapy was needed, were defined as the patients with an HFGS score equal to or more than 3 at nadir. The proportions of severe GBS patients with continuous CGSES scores ranging from 1 to 4 were analysed.

### Statistics

The Statistical Program for Social Science (SPSS, IBM, West Grove, PA, USA) version 18.0 was employed to accomplish the statistical analysis of all the data. For categorical variables, differences of proportion were analysed by Chi-square or Fisher exact test while qualitative variables by Mann-Whitney U test. The Student-t test was used to compare the values of normal continuous variables between two groups. Correlation between the values of two groups of normal continuous variable was tested by Pearson rank correlation coefficient (r) while between categorical variable and non-categorical variable by Spearman rank correlation coefficient (rs). The ordinal logistic regression was utilised to analyse the prognostic role of chief complaints, and the odds ratio (OR) was used to express the strength of prognostic effects. All tests were two-tailed, and the *p* value < 0.05 was set as the level of significance.

## Results

### Chief complaints are composed of seven elements

The clinical parameters of enrolled patient are presented in Table 1. Seven basic elements including weakness, numbness, pain, cranial nerve involvement, dyspnea, ataxia and autonomic dysfunction were found to compose the chief complaints of GBS patients. The details are provided in Table 2. In addition, 125 (23.90%) out of 523 patients reported these distinct kinds of symptoms with combinations thereof. Seventy patients complained of weakness and paraesthesia (including numbness and pain), 19 of weakness and cranial nerve involvement, 2 of weakness and autonomic disturbance, 12 of paraesthesia and autonomic dysfunction, 8 of paraesthesia and cranial nerve deficits as well as 1 of autonomic disorder and cranial nerve damage. The chief complaints of 13 GBS patients comprised at least 3 different kinds of elements, among whom 1 patient complained of four kinds.

**Table 1:** Demographics**, **clinical manifestations and laboratory findings of patients with Guillain-Barré syndrome (GBS)

Basic information	
Male/female ratio	311/212
Age (years), median (IQR)	36 (26–45)
Duration in hospital (days), median (IQR)	15 (10–20)
Symptoms of antecedent infection (*n* = 523)	
Interval between infection and onset (days), median (IQR)	5 (2–9)
Diarrhea, No. (%)	137 (26.20)
Upper respiratory tract infection, No. (%)	153 (29.25)
Severity of disease (*n* = 523)	
MRC sum score at admission, median (IQR)	48 (42–56)
HFGS at admission, median (IQR)	3 (2–4)
MRC sum score at nadir, median(IQR)	48 (36–54)
HFGS at nadir, median (IQR)	3 (2–4)
Interval between onset and nadir (days), median (IQR)	8 (6–12)
MRC sum score at discharge, median (IQR)	56 (49–60)
HFGS at discharge, median(IQR)	2 (1–3)
Clinical manifestations (*n* = 523)	
Cranial nerve involvement, No. (%)	236 (45.12)
Hyporeflexia/areflexia, No. (%)	484 (92.54)
Superficial sensation deficits, No. (%)	223 (43.05)
Autonomic deficits, No. (%)	289 (55.26)
Dyspnea, No. (%)	134 (25.62)
Ventilator dependence, No. (%)	64 (12.24)
Blood routine examination at admission (*n* = 523)	
White blood cell, mean (SD), 10^9^/L	9.00 (3.61)
Neutrophil, mean (SD), %	68.75 (3.63)
Lymphocyte, mean (SD), %	23.36 (0.10)
Lumbar puncture (*n* = 287)	
Protein concentration, mean (SD), g/L	1.05 (0.88)
White blood cell, mean (SD), 10^6^/L	3 (2–7)
Albumin-cytologic dissociations, No. (%)	192 (66.90)
IgG concentration, mean (SD), mg/L	189.24 (205.63)
Nerve conduction studies (*n* = 165)	
Demyelinating group, No. (%)	89 (53.94)
Axonal group, No. (%)	46 (27.88)
Overlap group, No. (%)	30 (18.18)

**Table 2:** Chief complaints in Guillain-Barré syndrome (GBS) patients

Chief complaint	Number of patients	Details, No. (%)
Weakness	384	Four limbs 294 (76.56), lower extremities 47 (12.24), upper extremities 20 (5.21), hands and feet 2 (0.52), hands 4 (1.04), asymmetric weakness 17 (4.43)
Numbness	161	Four limbs 100 (62.11), lower extremities 12 (7.45), upper extremities 6 (3.73), hands and feet 14 (8.70), hands 6 (3.73), feet 2 (1.24), fingers 4 (2.48), toes 1 (0.62), fingers and toes 1 (0.62), face 1 (0.62), whole body 1 (0.62), tongue 1 (0.62), tongue and face 1 (0.62), asymmetric numbness 1 (0.62)
Pain	19	Details were missing.
Cranial nerve involvement	73	Oculomotor and/or abducent nerve 25 (34), facial nerve 17 (23), glossopharyngeal and vagus nerve 35 (48), trigeminal nerve 2 (3)
Dyspnea	10	
Ataxia	2	
Autonomic dysfunction (including pain)	23	Pain 19 (83), palpitation 1 (4), urinary retention 1 (4), sweating 1 (4), swelling on hands 1 (4).

### Doctors find more than patients report

**Fig. 2:**
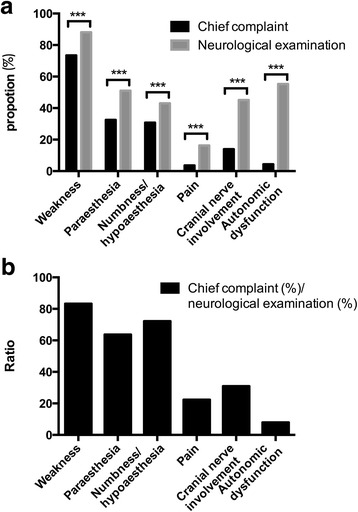
Neurological examinations and chief complaints. **a** Weakness, paraesthesia (including numbness/hypoaesthesia and pain), numbness/hypoaesthesia, pain, cranial nerve involvement, and autonomic dysfunction (including pain) were reported by 384 (73.42%), 170 (32.50%), 161 (30.78%), 19 (3.63%), 73 (13.96%) and 23 (4.40%) of 523 Guillain-Barré syndrome (GBS) patients. After neurological examination, doctors demonstrated that the symptoms mentioned above occurred in 461 (88.15%), 267 (51.05%), 223 (43.05%), 85 (16.25%), 236 (45.12%), and 289 (55.26%) of 523 GBS patients. Significant differences were observed for all the symptoms (*p* < .001). **b** The proportion was calculated by chief complaint (%)/neurological examination (%). It revealed that 83.29%, 63.67%, 72.19%, 22.34%, 30.93% and 7.96% of the GBS patients presented with weakness, paraesthesia, numbness/hypoaesthesia, pain, cranial nerve involvement, ataxia and autonomic dysfunction recognised these symptoms and contained them in their chief complaints. *p < 0.05, **p < 0.01, *** *p* < 0.001

A comparative study was conducted between the results of neurological examinations and the chief complaints (Fig. 2). Neurologists tended to find more symptoms after performing a careful physical examination than GBS patients initially demonstrated. For all the symptoms investigated including weakness, paraesthesia, cranial nerve involvement and autonomic dysfunction, significant differences were observed (Fig. 2). Weakness was not only the most frequent clinical manifestation, but also the most recognisable symptom by GBS patients. About 383 (73.42%) of 523 patients noticed weakness in their limbs, but neurologists observed that weakness occurred in 461 (88.15%) of all patients. Thus, only 383 (83.29%) of 461 patients could recognise the weakness that disturbed their daily lives and drove them to the doctors. In contrast, autonomic dysfunction was the most ignorable presentation; only 23 (4.40%) out of 523 patients complained of it, while neurologists actually identified it in 289 (55.26%) of these patients.

### Chief complaints aid prediction of disease severity and clinical manifestations

The prognostic role of weakness, numbness and cranial nerve involvement, which were among the most frequent chief complaints of GBS patients, was explored. The severity of GBS was related to the chief complaints of patients. Weakness is the most common symptoms among GBS patients [1], and MRC sum score and HFGS are designed to evaluated the disease severity based on strength deficits [13, 14]. It was not surprising that the patients with the chief complaint of weakness had significantly lower MRC sum scale score and higher HFGS at admission and nadir, while those with the chief complaint of numbness had significantly higher MRC sum scale score and lower HFGS at admission and nadir (Table 3).

**Table 3:** Relation between chief complaints and clinical features

	Complaint with/without weakness			Complaint with/without numbness		
	with	Without	*P* value	with	without	*P* value
Basic information						
Number of patient	384	139		161	362	
Male/female ratio	234/150	77/62	.23	90/71	222/140	.23
Age, median (IQR)	39 (29–48)	47 (34.25–58.75)	.042	41 (31–56)	40 (29.5–50)	.30
Antecedent infection						
Diarrhea, No. (%)	111 (28.80)	27 (19.59)	.031	36 (22.64)	100 (27.75)	.21
Upper respiratory tract infection, No. (%)	103 (26.93)	49 (35.14)	.063	64 (39.62)	90 (24.73)	.001
Clinical manifestations						
Cranial nerve involvement, No. (%)	152 (39.49)	79 (56.72)	< .001	82 (50.76)	157 (43.26)	< .001
Hyporeflexia, No. (%)	361 (94.03)	119 (85.96)	.002	99 (61.24)	134 (37.02)	< .001
Sensory deficits, No. (%)	160 (41.67)	65 (46.58)	.34	94 (58.22)	132 (36.39)	< .001
Autonomic deficits, No. (%)	200 (52.00)	88 (63.51)	.017	97 (60.38)	192 (53.02)	.14
Dyspnea, No. (%)	101 (26.40)	33 (23.65)	.16	32 (20.13)	101 (28.02)	.053
Ventilator dependence, No. (%)	46 (12.00)	18 (12.84)	.79	14 (8.81)	46 (12.74)	.11
Severity of disease						
MRC sum score at admission, median (IQR)	45 (32.75–52)	47 (37.5–56)	< .001	48 (42–56)	42.5 (31.25–51.5)	< .001
HFGS at admission, median (IQR)	4 (2.75–4)	3 (1.75–4)	< .001	3 (2–4)	4 (2–4)	< .001
MRC sum score at nadir, median (IQR)	42 (28–48.25)	42 (34–54.5)	< .001	46 (36–54)	36 (21–48)	< .001
HFGS at nadir, median (IQR)	4 (3–4)	3.5 (2–4)	.001	3 (3–4)	4 (3–4)	< .001
MRC sum score at discharge, median (IQR)	54 (44–57.25)	54 (42–60)	.002	55 (48–60)	48.5 (36.5–58)	< .001
HFGS as discharge, median (IQR)	2 (2–4)	1 (0–1.25)	.002	2 (1–3.75)	3 (1–4)	.009

Furthermore, chief complaints were related to the clinical features of GBS patients. A significantly large proportion of GBS patients complaining weakness had younger age, antecedent infection of diarrhea, decreased reflexes, but had less possibility to develop cranial nerve involvement and autonomic deficits during the hospitalisation than patients without this chief complaint (Table 3). Patients reporting numbness were more likely to have antecedent infection of upper respiratory tract infection and were more easily to develop cranial nerve involvement, hyporeflexia and sensory deficits (Table 2). Other negative results are also showed in Table 3.

Among 73 patients who were complaining of symptoms associated with cranial nerve involvement in clinic, such as dysphagia, ptosis and diplopia, cranial nerve involvement, dyspnea and ventilator dependence were more frequently observed than in 450 patients without the chief complaint of cranial nerve involvement [69 (93.85%) vs 172 (38.21%), *p* < .001; 28 (38.46%) vs 91 (20.31%), *p* = .001; 15 (20.00%) vs 44 (9.83%), *p* = .014]. GBS patients with the chief complaint of cranial nerve involvement also had significantly higher MRC sum scores/ lower HFGS at admission, nadir and discharge [MRC sum score, median (IQR): 54 (44–60), 48 (34.5–60), 58 (48–60) vs 44 (36–51), 40 (25–48), 50 (41.5–56), *p* < .001, < .001, < .001; HFGS: 3 (1–4), 3 (1–4), 1 (1–3) vs 3 (3–4), 4 (3–4), 3 (1–4), *p* < .001, .027, < .001].

### Chief complaints are predictors for prognosis

The short-term outcome of GBS was analysed at discharge and 6 months after discharge, respectively. The disease severity at discharge described by the MRC sum score and HFGS could be predicted by chief complaints. Patients with the chief complaint of weakness had significantly lower MRC sum scale score and higher HFGS at discharge, while with the chief complaint of numbness had significantly higher MRC sum scale score and lower HFGS at discharge (Table 3).

**Fig. 3:**
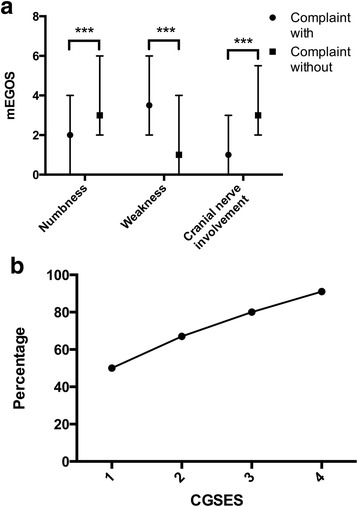
The predictive role of chief complaints. **a** The Guillain-Barré syndrome (GBS) patients complaining of weakness had poor outcomes 6 months after discharge (modified Erasmus GBS outcome scores (mEGOS): 3.5 with IQR of 2–6 vs 1 with IQR of 0–4, *p* < .001). However, the patients with the chief complaints of numbness and cranial nerve involvement were observed to have better prognosis (mEGOS: 2 with IQR of 0–4 vs 3 with IQR of 2–6, *p* < .001; 1 with IQR of 0–3 vs 3 with IQR of 2–5.5, *p* < .001). **b** The numbers (percentage) of severe GBS patients, with clinic GBS severity evaluation scale (CGSES) scores ranging from 1 to 4, were 10 (50%) (*n* = 20), 66 (67%) (*n* = 98), 79 (80%) (*n* = 98), and 136 (91%) (*n* = 150), respectively

In addition, the prognosis 6 months after discharge was evaluated by mEGOS. The median of mEGOS of all enrolled patients was 2, with IQR of 1 to 4. GBS patients with weakness as the chief complaint had a significantly higher mEGOS, while numbness and cranial nerve involvement a lower one (Fig. 3a). After preforming ordinal logistic regression, none of chief complaints of weakness, numbness or cranial nerve involvement lost the prognostic role (*p* = 0.016, 0.006 and 0.005), and the OR were 1.61, 0.60, and 0.49, respectively.

### Establishment of CGSES

Except for chief complaints of weakness, numbness and cranial nerve involvement, the duration between onset and admission was also included as a variable in the model due to its correlation to HFGS at nadir. The median of the duration between onset and admission for all GBS patients was 5 days with IQR of 3 to 8, and the duration was negatively correlated to the HFGS at nadir (*p* < .001, rs = −0.264). The CGSES was presented in Table 4A. Among 373 GBS patients, 7 patients were ruled out because of the missing of data. The percentages of severe GBS patients with the sum of the score ranging from −1 to 4, were 10 (50.00%), 20 (66.67%), 46 (67.65%), 79 (80.61%), 90 (89.11%) and 46 (93.88%), respectively. The patients scored −2 were not available. As the percentages of severe patients with the score of 0 and 1, 3 and 4 were close to each other, a converted score, which was defined as CGSES, was set in Table 4B. The percentages of severe patients with CGSES score ranging from 1 to 4 were presented in Fig. 3b. The median of the CGSES of 366 patients was 3 with the IQR of 2 to 4, and the CGSES score was positively correlated to the HFGS at nadir (*p* < .001, rs = 0.283).

**Table 4:** Clinic Guillain-Barré syndrome severity evaluation scale (CGSES)

Predictors					Score		
A.							
Chief complain of weakness							
with					1		
without					0		
Chief complain of numbness							
with					−1		
without					0		
Chief complain associated with cranial nerve involvement							
with					−1		
without					0		
Duration between onset and admission (days)							
0–2					3		
3–5					2		
6–10					1		
> 10					0		
sum of score					−2 to 4		
B.							
sum of score	−2	−1	0	1	2	3	4
CGSES	1	1	2	2	3	4	4

To validate our model, the data of the residual 150 patients were used. Seven patients with missing data were excluded. The percentages of severe GBS patients with CGSES scores ranging from 1 to 4 were 13 (53.85%), 45 (68.89%), 38 (78.95%) and 47 (91.49%), respectively. All of the values were within the 95% confidence intervals of the established model.

## Discussion

We explored the role of the chief complaint to predict the severity and outcome of GBS, as well as how the chief complaint may guide treatment options. Although various components are available in the chief complaints of GBS patients, weakness, numbness and cranial nerve involvement were the most popular. The chief complaint only covers a part of the clinical manifestations, and some existing symptoms are not chief complaints. Chief complaints are associated with clinical parameters, and are predictors for disease severity, clinical manifestations and prognosis. CGSES score, which is able to be evaluated after a quick inquiry in clinic, has a potential role to predict the disease severity at nadir and to guide the treatment option.

Results of physical examination were widely used to depict the feature of GBS and to evaluate the efficiency of treatment [1, 9, 15]. However, the chief complaint, has not drawn enough attention in most of the studies on the clinical features of GBS [1, 2]. According to the results of our study, some new concerns of GBS have arisen. Firstly, our study has listed chief complaint as the candidate for the predictors for GBS disease course. A number of studies have focused on the predictors for the disease course of GBS [6–10]. However, the chief complaint of GBS only appears in some case reports to provide information of special individuals [16, 17], and retrospective studies to unravel the prognostic role of the chief complaint are missing. Our results demonstrated that weakness, numbness and cranial nerve involvement were associated the severity and prognosis of GBS. Thus, chief complaint could assist clinicians to predict the disease course and the outcome of GBS at admission. As the MRC sum score and HFGS were designed based on the strength deficits among GBS patients, the chief complaint of weakness could be an important indicator for a severe disease course. When compared to the results of neurological examination, chief complaint is an easier and earlier obtained index to predict clinical severity and treatment options. A detailed neurological examination will be performed when the patients is hospitalised, and the results of the physical examination could imply the disease course. As a detailed examination is hardly available in clinic, chief complaint will be a more convenient parameter for clinicians to judge the severity of disease, especially to early recognise the risk of developing dyspnea. Secondly, the results of our study imply that a type of “latent GBS” may exist without notation. The results of our study demonstrate that some patients may not recognise all their symptoms, and they tended to report only symptoms that obviously disturbed their life. Thus, a latent form of GBS presenting as a period time of mild weakness may be out of the view of the neurologists. Patients may not go to hospital or not report it to doctors because of the mildness of disease, so the missed diagnosis occurs at outpatient clinic. With this consideration, clinicians should proactively ask about weakness when the patients report paraesthesia or report symptoms indicating cranial nerve or autonomic nerve involvement to avoid a missed diagnosis at outpatient clinic [2]. It is thus necessary to perform the paresis test on patients with a suspect of GBS. Last but not the least, CGSES had a potential role to guide the treatment option. The treatment of GBS is a combination of supportive care and immunotherapy. The immunotherapy is recommended when the HGFS score is equal to or more than 3 [18]. Over 80% of the GBS patients with CGSES score of 3 or 4 had an HGFS score equal to or more than 3 at nadir. Thus, we recommend that the immunotherapy may be used at admission on patients with a CGSES score of 3 or 4, especially 4, aiming at improving the prognosis.

Our study has limitations. Retrospective studies could be less reliable than prospective studies since validation of retrospectively collected data is complicated. However, in the current study, 523 GBS patients have been enrolled, and such a large sample volume could kind of guarantee the validation of the conclusions. A few patients with acute-onset chronic inflammatory demyelinating polyradiculoneuropathy might be enrolled in the study [19]. Due to the inadequate follow-up data and electrophysiological data, the associations between chief complaints and long-term outcome/subtypes of GBS were not well explored. The features of GBS patients reporting ataxia, dyspnea and autonomic dysfunction were not investigated due to the small sample volume.

With these concerns, more attention and studies are warranted to disclose the incidence and features of mild form of GBS, as the characteristics for mildly and severely affected GBS patients are dissimilar, regarding age, sex and antecedent infection [14]. The role of the chief complaints of ataxia, dyspnea and autonomic dysfunction should be further explored. Studies are also warranted to explore whether distinct chief complaints indicate different immune response of GBS patients. CGSES score need to be further confirmed in prospective studies as well.

## Conclusions

Weakness and numbness are the most common chief complaints among GBS patients. Chief complaints are predictors of disease severity, ventilator dependence and short-term outcome. Immunotherapy may be used at admission on patients with a CGSES score of 3 or 4.

## Abbreviations

CGSES: Clinic GBS severity evaluation scale
GBS: Guillain-Barré syndrome
HFGS: Hughes functional grading scale
IVIg: Intravenous immunoglobulin
mEGOS: Modified erasmus GBS outcome score
MRC: Medical research council
OR: Odds ratio
